# The Hierarchical Brain Network for Face Recognition

**DOI:** 10.1371/journal.pone.0059886

**Published:** 2013-03-20

**Authors:** Zonglei Zhen, Huizhen Fang, Jia Liu

**Affiliations:** 1 State Key Laboratory of Cognitive Neuroscience and Learning, Beijing Normal University, Beijing, China; 2 Institute of Psychology, Chinese Academy of Sciences, Beijing, China; University of Montreal, Canada

## Abstract

Numerous functional magnetic resonance imaging (fMRI) studies have identified multiple cortical regions that are involved in face processing in the human brain. However, few studies have characterized the face-processing network as a functioning whole. In this study, we used fMRI to identify face-selective regions in the entire brain and then explore the hierarchical structure of the face-processing network by analyzing functional connectivity among these regions. We identified twenty-five regions mainly in the occipital, temporal and frontal cortex that showed a reliable response selective to faces (versus objects) across participants and across scan sessions. Furthermore, these regions were clustered into three relatively independent sub-networks in a face-recognition task on the basis of the strength of functional connectivity among them. The functionality of the sub-networks likely corresponds to the recognition of individual identity, retrieval of semantic knowledge and representation of emotional information. Interestingly, when the task was switched to object recognition from face recognition, the functional connectivity between the inferior occipital gyrus and the rest of the face-selective regions were significantly reduced, suggesting that this region may serve as an entry node in the face-processing network. In sum, our study provides empirical evidence for cognitive and neural models of face recognition and helps elucidate the neural mechanisms underlying face recognition at the network level.

## Introduction

The ability to quickly and accurately recognize faces is arguably one of the most developed visual skills in humans. To investigate the neural mechanisms underlying this fascinating ability, numerous functional magnetic resonance imaging (fMRI) studies have identified multiple cortical regions that show a higher response for faces than for non-face objects [1]–[5]. The most frequently localized regions are in the occipitotemporal cortex, such as those in the fusiform gyrus (FG, or fusiform face area, FFA) [6], [7], inferior occipital gyrus (IOG, or occipital face area, OFA) [8], [9] and posterior superior temporal sulcus (pSTS) [10], [11]. These three regions are thought to constitute the core system for face recognition[2], [12]: FG and IOG analyze invariant aspects of faces that underlies recognition of individuals[10], [13], [14], whereas pSTS processes the changeable aspects of faces such as the direction of eye gaze, facial expression and lip movements for facilitating social communications [10], [11], [15]. In addition, face-selective regions beyond the occipitotemporal cortex have been observed. For example, the amygdala and insula are tuned to emotional aspects of facial expression [16]–[20]; a region in the intraparietal sulcus (IPS) is activated when the direction of eye gaze shifts spatial attention [10], [11]; regions located in the temporal pole (TP) and anterior middle temporal gyrus (aMTG) are sensitive to the familiarity of faces [21]–[23]; a region in the inferior frontal gyrus (IFG) is involved in processing the semantic aspects of faces [22], [24]; and a region in the orbital frontal cortex (OFC) is involved in extracting information on facial beauty [25]–[27].

However, many previous studies focus on the functional profile of one individual face-selective region, not the properties of the face-processing network constituted by these regions. Yet, typical face recognition depends not only on the intact functionality of individual regions, but also the dynamic interaction among them [28]–[32]. In this study, we asked how these face-selective regions constitute a hierarchically structured face-processing network through synchronized neural activation among them, henceforth called functional connectivity. To this end, we first localized face-selective regions in the entire brain that served as nodes for network-level analyses. Specifically, face-selective regions were identified for each participant guided by a group-level probabilistic map of face-selective activation [33], [34]. Second, the reliability and selectivity of these regions were evaluated to ensure that they were truly involved in face processing. Third, the hierarchical structure of the face-processing network constituted by these regions was characterized on the basis of the strength of the functional connectivity among them. Finally, we examined the dynamic property of the face-processing network when participants switched tasks between face recognition and object recognition.

## Methods

### Participants

Forty-two college students (aged 20–30 years; 18 females) participated in the study. All participants were right-handed and had normal or corrected-to-normal visual acuity. Ten participants were scanned seven times over seven consecutive days (i.e., seven scan sessions in total), and the rest of the participants were scanned once. The fMRI protocol was approved by the Institutional Review Board of Beijing Normal University, Beijing, China. Written informed consent was obtained from all participants before the experiment.

### Experimental Procedure

In each session, two blocked-design functional localizer runs were conducted. Each run consisted of blocks of human frontal-view faces, familiar objects, scenes and scrambled objects. Scrambled objects were generated by superimposing a grid over object images and then relocating the component squares randomly. Each run lasted 5 min and 36 sec and consisted of sixteen 16-sec blocks (i.e., four blocks per condition) with five 16-sec fixation periods being interleaved. During each block, twenty exemplars of a given stimulus category were presented sequentially, each of which was presented for 300 ms in the center of the screen followed by a blank interval of 500 ms. Participants pressed a button whenever two identical images were presented in a row (i.e., one-back task). The task was designated to maintain roughly the same amount of attention among stimulus categories.

### fMRI Data Acquisition

Scanning was conducted on a Siemens 3T scanner (MAGENTOM Trio, a Tim system) with an eight-channel phased-array head coil at BNU Imaging Center for Brain Research, Beijing, China. The whole brain fMRI data were collected using a T2*-weighted gradient-echo, echo-planar imaging sequence (EPI) (TR = 2 sec, TE = 30 ms, FA = 90 degrees, matrix = 64×64, 25 slices, voxel size = 3×3×4 mm). In addition, MPRAGE, an inversion prepared gradient echo sequence (TR/TE/TI = 2.53 sec/3.45 ms/1.1 sec, FA = 7 degrees, voxel size = 1×1×1 mm), was used to acquire 3D structural images.

### fMRI Data Preprocessing

fMRI data analyses were performed with fMRI Expert Analysis Tool (FEAT) of FSL (FMRIB’s Software Library, http://www.fmrib.ox.ac.uk/fsl). Preprocessing was performed with the default parameters of FEAT, consisting of motion correction, brain extraction, high-pass temporal filtering (0.01 Hz cutoff), spatial smoothing with a Gaussian kernel (FWHM = 5 mm). Then, each run in a session was modeled separately for each participant. A boxcar was convolved with a gamma hemodynamic response function, and its temporal derivative was used to model blood oxygen level-dependent (BOLD) signal changes. Statistical analyses on time series were performed with FILM (FMRIB’s Improved Linear Model) with a local autocorrelation correction. The statistic image for each run was thresholded using clusters determined by Z>2.3 and a corrected cluster significance of *p*<0.05, assuming a Gaussian random field for the Z statistics. Finally, the statistic image from each run was registered to each participant’s high-resolution structural image, and then transformed to the standard MNI152 template by using FLIRT (FMRIB’s Linear Image Registration Tool) for group analyses.

### Localizing Face-selective Regions

The traditional approach in defining a region of interest (ROI) at the individual level is to select a set of activated voxels with the consideration of between-subject variance in structural anatomy [35], [36]. However, this approach is time-consuming and heavily relies on experimenters’ expertise in defining ROIs. In this study, we adopted a new method, called the group-constrained subject-specific (GSS) approach, to automatically define ROIs at the individual level [33], [34]. As its name suggests, the GSS approach uses a probabilistic map acquired at the group level to guide the selection of relevant voxels at the individual level.

In particular, face-selective regions were defined in four steps with the GSS approach in this study. First, the activation maps by the contrast of faces versus objects of all participants from the first run of the first scan session were overlaid onto the MNI152 template to generate a probabilistic map. The value for a voxel in the map was the number of participants who showed a significantly higher response for faces than for objects at this voxel (Z>2.3, cluster-corrected significance threshold *p*<0.05). The value, therefore, provided an index for the consistency of activation at the voxel level. Second, the probabilistic map was smoothed using a Gaussian kernel (FWHM = 6 mm) to eliminate spurious local maxima. Then, the smoothed map was segmented into anatomically separated regions by using a watershed algorithm [37]. The watershed algorithm is a region-based segmentation approach, an analogy of a landscape being flooded by water. That is, water fills up catchment basins from the local minima to the highest peak. During this process, water coming from different basins meets at watershed lines, and the landscape is partitioned into multiple regions separated by the watersheds. Here, the probabilistic map was first flipped by multiplying −1, and was then treated as the landscape in the watershed algorithm. That is, the local minima of the landscape (i.e., the catchment basins) corresponded to the local maximum of the probabilistic map, and the watershed lines were the borders among face-selective regions. As a result, a set of group-level ROIs were generated from the partition. The percentage of participants who had at least one significantly activated face-selective voxel within the ROI provided an index for the consistency of activation at the ROI level. Of note, the value for the consistency of activation at the ROI level was in general larger than the value at the voxel level, because the former did not differentiate whether face-selective voxels within the ROI were overlapping or not. Third, a group-level ROI was removed if it consisted of only a small portion of participants who showed face-selective voxels in the ROI. The criterion was set to 60% (i.e., at least 60% of participants who had the face-selective voxels in the ROI) to balance the need to localize as many ROIs as possible for network analyses with the need to localize ROIs in as many participants as possible. Finally, the group-level ROIs were intersected with each individual’s activation map to generate subject-specific ROIs. That is, the group-level ROIs were used to constrain the selection of subject-specific ROIs.

### Evaluating Reliability and Selectivity of the Face-selective ROIs

Except those well-studied regions such as FG, IOG and pSTS, the ROIs localized above may not be truly face-selective. Therefore, before the ROIs were used to construct the face-processing network, their reliability and selectivity were examined with an independent set of data. The reliability analysis consisted of cross-subject reliability and cross-session reliability. The selectivity analysis examined whether the selectivity established by the contrast of faces versus objects could be generalized to other non-face objects (e.g., scenes). The scrambled object condition was designated to localize object-selective regions (i.e., objects versus scrambled objects), and therefore it was not used in the present study on the face-processing network.

#### Cross-subject reliability

In this component, we examined whether the ROIs defined in the first run retained their selectivity for faces in the second run. Specifically, the percent BOLD signal changes for faces and objects in the second run were extracted from the ROIs defined in the first run for each participant. Pair-wise *t*-tests were conducted to test whether the response for faces was significantly higher than that for objects. The criterion for cross-subject reliability was set to a significance level of *p*<0.05(FDR corrected). ROIs that failed to meet this criterion were removed.

#### Cross-session reliability

In this step, we examined whether the ROIs defined in one session could be reliably localized in multiple scan sessions from ten participants who were scanned once daily for seven consecutive days. An ROI was considered face-selective in a session if there was at least one voxel in the ROI that showed a significantly higher response for faces than for objects. Like most power analyses, 80% was specified as the desired level of power to be achieved in cross-session reliability, as we expected that success (i.e., face-selective responses existed in a face-selective ROI) was four times as likely as failure in a session. That is, only when an ROI was found face-selective in 80% of all scan sessions did it meet cross-session reliability. ROIs that failed to pass the criterion were discarded.

#### Face selectivity

In this measurement, we examined whether the selectivity of an ROI defined by the contrast of faces versus objects can be generalized to other objects by comparing its response for faces to its response for scenes that were not used to define the ROI. Specifically, an ROI defined in the first run must meet two criteria for face selectivity in the second run. First, the response of an ROI for faces must be significantly higher than for the fixation baseline. Second, the response for faces must be significantly higher than for scenes that were not used to define the ROI in the first run. The criterion for face selectivity was set to a significance level of p<0.05(FDR corrected). ROIs that failed to meet this criterion were removed.

### Network Analyses on Functional Connectivity

After identifying face-selective ROIs, we investigated how they constituted the face-processing network through functional connectivity among them and what the dynamic nature of the network was when participants switched tasks between face recognition and object recognition.

#### Hierarchical clustering analysis

Here we used the strength of functional connectivity among the ROIs to characterize the hierarchical structure of the face-processing network. First, the time courses of the BOLD signals of all voxels within an ROI in each run were extracted and averaged across voxels. Second, to remove fluctuations from head motion, six parameters obtained by rigid body corrections for head motion with their temporal derivatives were regressed out from the averaged time course. Third, the residual time courses of all face blocks in the session from an ROI were normalized to *z* scores, which were then concatenated as one continuous time course. Because there were eight data points in a face block (i.e., 16 sec per block with TR being 2 sec), four face blocks in a run and two runs in a session, there were sixty-four data points in total in the time course of an ROI of a participant. Fourth, for each participant, a matrix on functional connectivity was created by calculating Pearson correlation coefficient (*r*) between the time courses of each pair of ROIs. The matrices were then averaged across participants. Then, a hierarchical cluster analysis with Ward linkage method [38] was applied to the averaged matrix to determine which pairs of ROIs were most synchronized and which were least synchronized. The value of “1– *r*” was used as an index for distance in the clustering. The resulting clusters, or dendrogram, were assessed by the cophenetic correlation coefficient, which is a measure of how faithfully the dendrogram represents the dissimilarities among observations [39]. Specially, the cophenetic correlation is defined as the linear correlation coefficient between original distances (i.e., dissimilarities) used to construct the dendrogram and cophenetic distances obtained from the dendrogram (i.e., the height of the link in the dendrogram at which observations are first joined). The more faithful the dendrogram is, the closer to 1 the cophenetic correlation coefficient is. The hierarchical clustering was considered successful if the cophenetic correlation coefficient was larger than 0.75. Finally, brain network was visualized with BrainNet Viewer (http://www.nitrc.org/projects/bnv/).

#### Dynamic properties of the network

To investigate how the face-processing network adapted to different computational demands, we compared the connectivity matrix obtained in the face-recognition task with that obtained in the object-recognition task. First, the connectivity matrix for the object task was calculated for each participant, similar to the aforementioned matrix for the face task. Then, pair-wise *t*-tests were used to examine which pairs of ROIs showed significant changes in functional connectivity when the task was switched from the face task to the object task. False discovery rate (FDR) was used to correct multiple comparisons at the significance level of *q* = 0.05.

## Results

### Twenty-five Face-selective Regions are Identified in the Entire Brain

Because there is considerable amount of variability in face-selective activation across individuals and across scan sessions [40]–[42], regions that are truly involved in face processing shall meet three criteria: (1) consistent anatomic location across individuals (cross-subject reliability), (2) replicable selectivity for faces across sessions within an individual (i.e., cross-session reliability), and (3) selective responses for faces but not for a variety of non-face objects.

**Figure 1:**
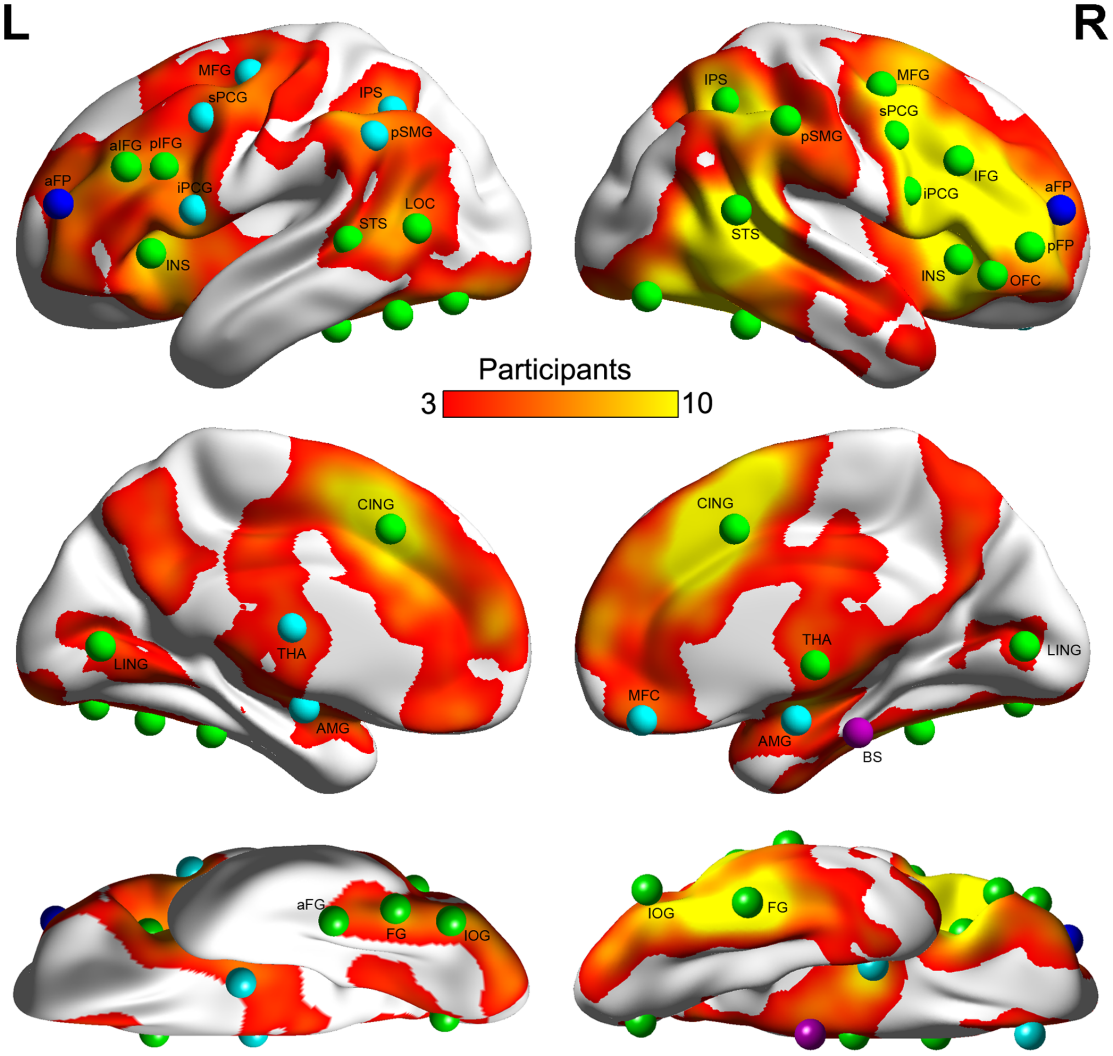
Probabilistic map and peak locations of group-level ROIs overlaid on mean MNI152 brain surface. The probabilistic map was created by overlaying participants’ binarized activation maps with the contrast of faces versus objects. Color bar indicates the number of participants who showed face-selective activation. Spheres indicate the peak locations of thirty-seven group-level ROIs. ROIs that failed to meet the criteria of cross-subject reliability, cross-session reliability and face selectivity were colored with magenta, blue and cyan, respectively. ROIs that met all criteria were colored with green. L: left hemisphere; R: right hemisphere.

To identify face-selective regions, the GSS approach (see Methods) was used to generate a probabilistic map that showed the degree of consistency across the participants in response to faces (versus objects) in the brain (Figure 1). In addition to its similarity to the statistical map from the traditional random-effect group analysis (Figure S1), the probabilistic map provided additional information on the consistency of face-selective activation across the participants, with the number of participants who showed face-selective activation at a voxel as an index for the consistency of activation at the voxel level. The voxel with highest consistency of activation (25 out of 42 participants) was located in the right FG (MNI coordinates: 44, −46, −23), encompassed in the right FFA as reported previously (e.g., [43]).

The probabilistic map was further segmented into 137 anatomically separated regions with a watershed algorithm. The percentage of participants who showed face-selective voxels within the ROI provided an index for the consistency of activation at the ROI level. Among them, only 37 regions held the consistency of activation higher than 60% (i.e., these regions contained face-selective voxels in more than 60% of the participants) (Figure S2).

These regions were widely distributed across the entire brain, with 32 regions in the cerebral cortex and 5 in the subcortical regions (Figure 1). These group-level regions were then intersected with each participant’s face-selective activation map to generate subject-specific ROIs for each participant (Figure S3). These ROIs in total consisted of 63.9% of all voxels showing face-selective activation in all participants. Next, we examined the reliability and selectivity of these 37 ROIs with an independent dataset.

The evaluation of cross-subject reliability was performed by examining whether the face selectivity of an ROI defined across participants in the first run remained in the second run. We found that 36 out of 37 ROIs showed a significantly higher response for faces than for objects in the second run (all *p*<0.05, FDR corrected). The region that failed this criterion was located in the brain stem (BS) (the magenta-colored sphere in Figure 1). In addition, we examined the cross-session reliability of the rest of the ROIs in the participants who were scanned seven times on separate days. We found that 34 out of 36 ROIs showed face-selective activation in more than 80% of the total scan sessions. Two regions that failed this criterion were located in the anterior portion of both the frontal poles (L and R aFP) (the blue-colored spheres in Figure 1). By contrast, regions in the bilateral FG (L and R FG), right superior temporal sulcus (R STS), right superior portion of the precentral gyrus (R sPCG) and right IFG (R IFG) were reliably localized in all scan sessions and in all participants.

In addition to the reliability of the ROIs, we examined whether the selectivity for faces in the ROIs can be generalized to objects that were not used to define the ROIs. We observed that ROIs in the bilateral amygdala (L and R AMG), posterior portion of the left supramarginal gyrus (L pSMG), and right medial frontal cortex (R MFC) failed to show a significantly higher response for faces than for the fixation baseline. In addition, ROIs in the left superior and inferior precentral gyrus (L sPCG and L iPCG), left intraparietal sulcus (L IPS), the left middle frontal gyrus (L MFG) and left thalamus (L THA) did not show a significantly higher response for faces than for scenes (the cyan-colored spheres in Figure 1) (See Figure S4 for the magnitude of responses).

The remaining 25 regions satisfied both the reliability and selectivity criteria (the green-colored spheres in Figure 1) (Table 1 and Figure S5). They were distributed throughout the brain and were approximately symmetrically located in both hemispheres, with the total size of the ROIs in the right hemisphere (RH: 5.62×10^4^ mm^3^) being about two times larger than that in the left hemisphere (LH: 2.75×10^4^ mm^3^). Next, we characterized the hierarchical structure of the face-processing network comprised of these 25 face-selective regions through functional connectivity.

**Table 1:** The group-level and individual-level face-selective ROIs: reliability, selectivity, and coordinates.

Lobe	ROI name	Percent subjects	Group -level ROI size (mm^3^)	Avg. GSS ROI size (mm^3^)	Location of peak overlap (MNI)	Faces>objects (p-value)	Faces>fixation(p-value)	Faces>scenes(p-value)	Avg. cross-session reliability
**Temporal**	L aFG	62%	792	82	−38 −38 −24	<0.0001	<0.0001	<0.0001	0.857
	L STS	71%	1398	165	−54 −42 4	<0.0001	<0.0001	<0.0001	0.886
	L FG	98%	2367	238	−42 −58 −20	<0.0001	<0.0001	<0.0001	1
	R STS	98%	8256	1443	56 −49 14	<0.0001	<0.0001	<0.0001	1
	R FG	95%	5851	923	44 −46 −23	<0.0001	<0.0001	<0.0001	1
**Occipital**	L IOG	76%	1263	168	−38 −76 −15	<0.0001	<0.0001	<0.0001	0.9
	L LOC	93%	3403	382	−48 −64 8	<0.0001	<0.0001	<0.0001	0.986
	R IOG	81%	2047	267	48 −78 −14	<0.0001	<0.0001	<0.0001	0.986
	L LING	86%	4393	578	−7 −78 −4	<0.0001	<0.0001	0.001	0.929
	R LING	76%	2548	279	6 −80 −1	<0.0001	<0.0001	0.039	0.857
**Parietal**	R IPS	86%	6627	1041	32 −52 49	<0.0001	<0.0001	<0.0001	0.957
	R pSMG	69%	1426	309	62 −33 43	<0.0001	<0.0001	0.001	0.871
**Frontal**	L aIFG	74%	2092	338	−45 30 28	<0.0001	0.004	0.001	0.843
	L pIFG	71%	927	134	−42 18 28	0.003	0.036	0.042	0.814
	L CING	95%	7162	955	−2 20 42	<0.0001	<0.0001	0.001	0.957
	R OFC	74%	1369	312	47 34 −7	<0.0001	0.003	0.001	0.843
	R pFP	81%	2632	489	44 46 2	<0.0001	<0.0001	<0.0001	0.943
	R IFG	93%	5457	1349	48 23 30	<0.0001	<0.0001	<0.0001	1
	R MFG	79%	2644	471	40 −2 54	<0.0001	<0.0001	<0.0001	0.929
	R CING	93%	7219	1210	2 14 42	<0.0001	<0.0001	<0.0001	0.957
	R sPCG	88%	2173	426	47 2 38	<0.0001	<0.0001	<0.0001	1
	R iPCG	95%	2221	421	51 6 20	<0.0001	<0.0001	<0.0001	0.957
	L INS	90%	3732	405	−34 22 0	<0.0001	<0.0001	<0.0001	0.971
	R INS	93%	2783	465	34 23 −2	<0.0001	<0.0001	<0.0001	0.914
**Subcortical**	R THA	81%	2931	337	2 −12 −2	0.028	0.028	0.001	0.929

FG, Fusiform Gyrus; LING, Lingual Gyrus; STS, Superior Temporal Sulcus; IOG, Inferior Occipital Gyrus; LOC, Lateral Occipital Cortex; IPS, Intraparietal Sulcus; SMG, Supramarginal Gyrus; IFG, Inferior Frontal Gyrus; CING, Paracingulate.

Gyrus; OFC, Orbital Frontal Cortex; FP, Frontal Pole; MFG, Middle Frontal Gyrus; PCG, Precentral Gyrus; INS, Insular.

Cortex; THA, Thalamus; a, anterior; p, posterior; s, superior; i, inferior; L, left; R, right.

### The Face-processing Network Consists of Three Sub-networks

**Figure 2:**
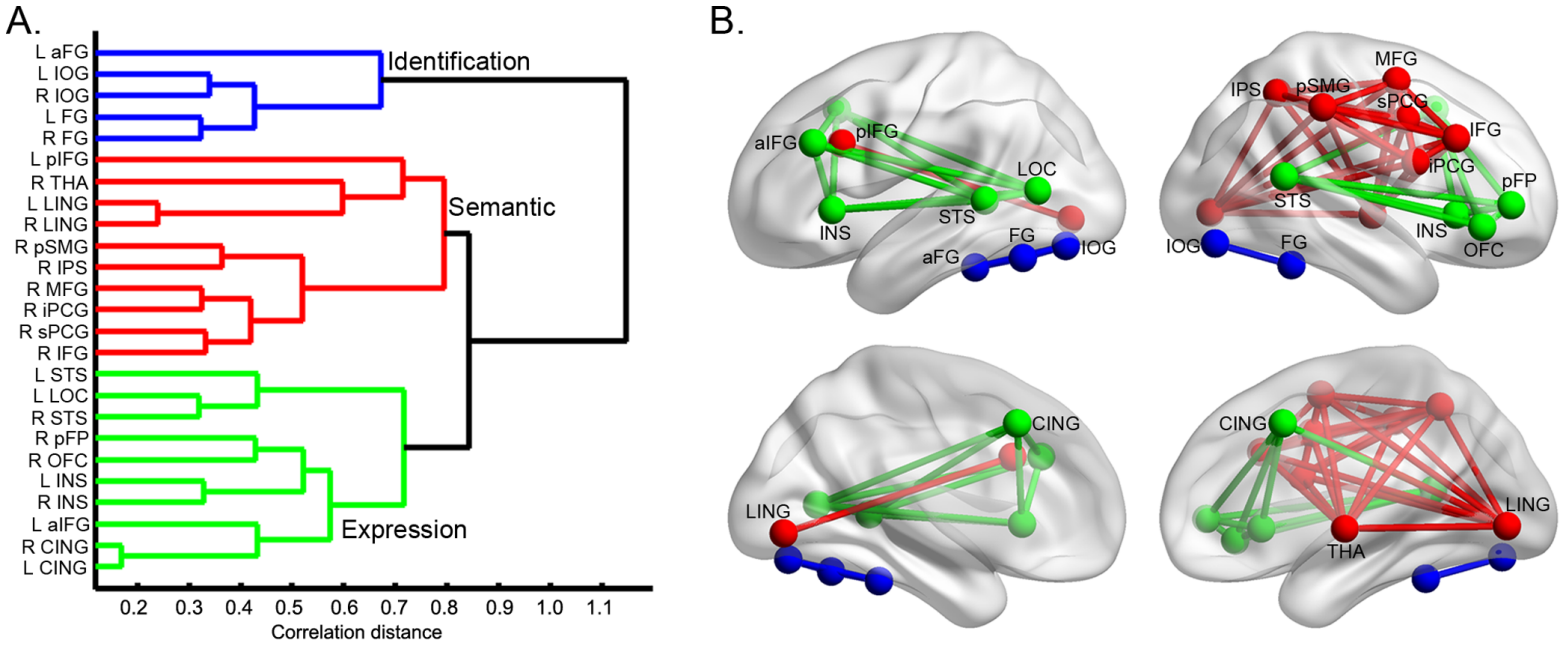
Hierarchically structured face-processing network. (A) Dendrogram from the hierarchy clustering analysis based on the strength of functional connectivity among the face-selective ROIs when the participants performed a face recognition task. The face-processing network consists of three relatively independent sub-networks that correspond to the recognition of individual identity (Identification), retrieval of personal knowledge (Semantic) and analysis of facial expression (Expression). (B) Sub-networks are displayed on the mean MNI152 brain surface with nodes and edges. The sub-network for Identification, Semantic, and Expression is colored with blue, red and green, respectively. Note that for display purposes, edges between the sub-networks are not shown.

The face-processing network was constructed on the basis of the strength of the functional connectivity among the ROIs when the participants performed a face-recognition task. Functional connectivity between a pair of ROIs was calculated as the temporal correlation between the time courses extracted from the ROIs. On average, the functional connectivity between all pairs of ROIs was relatively strong (mean ± standard deviation: 0.45±0.1). Importantly, the hierarchical clustering analysis on the functional connectivity matrix revealed that the face-selective ROIs were grouped into three relatively independent sub-networks (Cophenetic correlation coefficient = 0.86) (Figure 2A). ROIs in the occipital (L and R IOG) and temporal cortex (L FG, R FG and L aFG) formed the first sub-network (Figure 2B, blue). The second sub-network consisted of ROIs in the frontal cortex (L pIFG, R MFG and R IFG), precentral gyrus (R iPCG and R sPCG), parietal cortex (R IPS and R pSMG), lingual gyrus (L and R LING) and right thalamus (R THA) (Figure 2B, red). The rest of the ROIs in the frontal cortex (R pFP, R OFC and L aIFG), lateral occipital cortex (L LOC), superior temporal sulcus (L and R STS), paracingulate gyrus (L and R CING) and insular cortex (L and R INS) constituted the third sub-network (Figure 2B, green). In addition, the hierarchical clustering analysis revealed that both the distance between the identification and semantic sub-network and that between the identification and expression sub-network were larger than the distance between the semantic and expression sub-networks, suggesting that the identification sub-network is more distinct from the rest of the two sub-networks.

One possible interpretation of the hierarchical structure of the face-processing network is that it may result from anatomical distance between the face-selective regions. Because the strength of functional connectivity is inversely correlated with the anatomical distance between regions, it is expected that neighboring ROIs were clustered together (e.g., IOG and FG). However, the anatomical distance cannot fully account for the hierarchical structure of the face-processing network for three reasons. First, the interhemispheric pairs of homologous ROIs (e.g., L and R FG) showed strong long-range connectivity, and they were grouped into the same sub-network at the first level. Second, some intrahemispheric regions that are located in different lobes, such as the ROIs from the occipitotemporal cortex (e.g., STS and LOC) and from the frontal cortex (e.g., R pFP, R OFC and L aIFG) were clustered into the same sub-network. Finally, the hierarchical clustering analysis based on the anatomical (Euclidean) distance among these ROIs generated a qualitatively different set of sub-networks (Figure S6). Therefore, the hierarchical structure based on functional connectivity partly reflects the network-level property of the face-processing network in processing faces.

### IOG Serves as an Entry Node in the Face-processing Network

**Figure 3:**
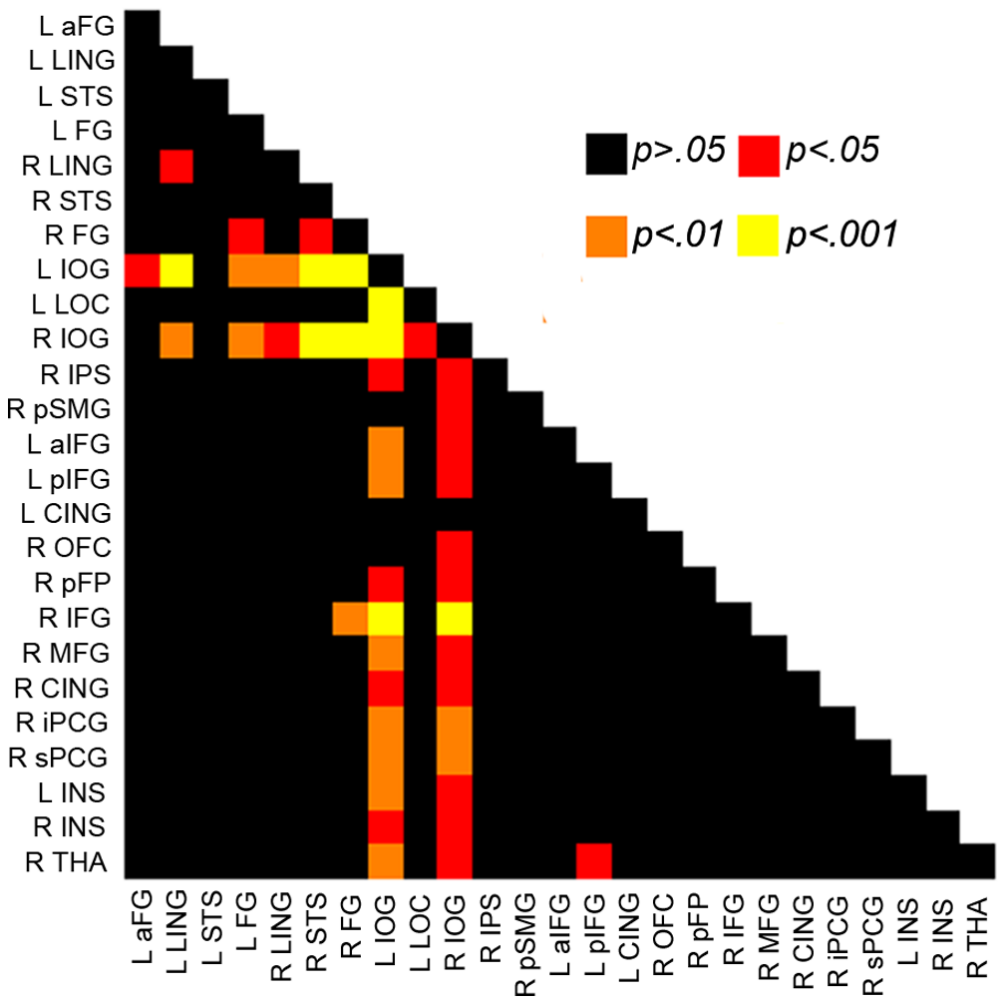
The IOG plays a pivotal role in the face-processing network. The matrix indicates changes in functional connectivity among the face-selective regions when the participants switched from the face-recognition task to the object-recognition task. Black cells indicate that the change in functional connectivity is not significant, whereas colored cells show that the functional connectivity between the face-selectivity regions is significantly reduced (*p*<0.05, FDR corrected). Note that the decrease in functional connectivity is mainly observed between the IOG (i.e., OFA) and the rest of the face-selective regions.

We further asked how the face-processing network dynamically adjusts its weights in functional connectivity among constituent nodes to adapt to different computational demands. To this end, a new functional connectivity matrix of the ROIs was generated in the object-recognition task in the same manner as that in the face-recognition task. Then, the functional connectivity matrix acquired in the object-recognition task was compared to that in the face-recognition task. That is, changes in functional connectivity were examined on each pair of ROIs when the task was switched from the face task to the object task. We found that the functional connectivity in the face-processing network in general was significantly reduced when the participants switched to the object-recognition task from the face-recognition task (FDR, *q*<0.05). Interestingly, the significant decrease in functional connectivity was mainly found between the IOG (i.e., OFA) and the rest of the ROIs. Specifically, 21 out of 24 ROIs showed reduced functional connectivity with the right IOG, whereas 20 out of 24 ROIs showed reduced connectivity with the left IOG (all *p*<0.05, FDR corrected) (Figure 3). In addition, the functional connectivity between the left and right LING, left and right FG, right FG and right STS, right IFG and right FG, and right THA and left pIFG was also reduced (all p<0.05, FDR corrected). No other significant decrease in functional connectivity was observed (all p>0.05). Interestingly, the significant changes in connectivity mainly occurred both within the identification sub-network and between the identification sub-network and the rest of two other two sub-networks because the only difference of the two tasks was the category of stimulus.

## Discussion

In this study, we characterized the face-processing network comprised of face-selective regions in the brain. We first identified twenty-five regions showing reliable face-selective activation across participants and across scan sessions. The functional connectivity analysis revealed that these regions were clustered into three relatively independent sub-networks. Importantly, the IOG may serve as an entry node of the face-processing network, as the functional connectivity between the IOG and the rest of the regions were significantly decreased when the participants switched from the face-recognition task to the object-recognition task. In short, our study provides some of the first empirical evidence of the face-processing network throughout the brain, inviting further studies on face recognition from the network perspective.

Nodes are the functional building blocks of brain networks. In addition to the well-studied face-selective regions in the occipitotemporal cortex, reliability and selectivity of the remainder of the face-selective regions must be evaluated before they may be considered as nodes functioning in the face-processing network. That is, a face-selective region should not only show a significantly higher response to faces (versus a variety of non-face objects) but also be reliably replicated across participants and across scan sessions. On the basis of these criteria, twenty-five regions were identified in the brain, comprised of nearly all of the face-selective regions previously identified [2], [34], [44], [45]. Interestingly, the massive cluster in the frontal cortex was found to contain multiple peaks, and thus was further divided into various smaller face-selective regions (e.g., IFG, sPCG, iPCG, OFC and pFP). Furthermore, the functional connectivity analysis revealed that these frontal regions belonged to different sub-networks. Future work is required to elucidate the functional divisions of labor among these regions in the frontal cortex. In contrast, regions in the amygdala and anterior temporal lobe that were previously identified as face-selective regions did not meet the selectivity criterion in the current study, possibly because we used novel faces with neutral expressions [16], [18], [20].

The functional connectivity analysis revealed that these face-selective regions were organized in a hierarchical structure with three sub-networks. The first sub-network consisted of the IOG and FG, presumably involved in recognizing face identity [10], [13], [14]. Regions such as the MFG and IFG formed the second sub-network, possibly involved in accessing semantic information contained in faces [22], [24]. The third sub-network was constituted by the regions that seem tuned to facial expression, such as the STS, OFC, and INS [16], [17], [20]. These three sub-networks derived from the functional connectivity analysis extend the neural model proposed by Haxby and colleagues (2000) [2] (see also Bruce and Young, 1986 [46]). The first sub-network identified in this study corresponds to the core system in the model that is engaged in representing invariant aspects of faces to discriminate individuals. Importantly, our study demonstrates that the extended system in the model can be further divided into two sub-systems, one for processing facial expressions and the other for analyzing semantic information associated with faces.

However, the three face sub-networks are not completely independent; instead, face-selective regions in one sub-networks may possess functions of another sub-network. For example, the FG in the identification sub-network also shows a higher response for expressive faces than neutral faces [20], [47]–[50], while the STS in the expression sub-network shows an adaptation effect not only to facial expression but also to facial identity [51], [52]. The division of the face-processing network into three sub-networks does not contradict with these findings. First, although some face-selective regions may be involved in multiple functions, they show different levels of preferences in processing different aspects of faces. For example, the STS prefers to process facial expression, whereas the FG prefers to process facial identity [10]. Second, the hierarchal cluster analysis in this study was based on the relative, not absolute, separability of the face-selective regions, or their preferences in processing different aspects of faces. Therefore, the distinctions among the sub-networks are relative, and regions in one sub-network may be recruited to process faces jointly with regions in other sub-networks.

Interestingly, the functional connectivity in the face-processing network was generally reduced when the task was switched from face recognition to object recognition, suggesting that the face-processing network dynamically adjusts its weight in connectivity among the face-selective regions to adapt to different computational demands. Importantly, the IOG, which abuts the FG ventrally and STS dorsally, plays a pivotal role in dynamically adjusting the weights, as the functional connectivity between IOG and the rest of the face-selective regions was decreased after the task was switched from face recognition to object recognition. This observation is consistent with the previous finding that the IOG is activated around 100 ms after stimulus onset [53], [54] and then provides input to both the FG and STS [29], [30]. In addition, although IOG is specialized in processing faces, it processes faces in the parts-based fashion, similar to the manner in which non-face objects are processed [54]–[58]. Therefore, the IOG may serve as a bridge connecting two types of processing: the holistic processing of faces and the parts-based processing of objects, which makes it perfectly suitable as a critical node that connects the face-processing network and the network involved in processing non-face objects. However, this finding does not necessarily suggest that the IOG is the only node that the information flowing from the early visual cortex to the face-processing network. Instead, previous studies have suggested the existence of other possible pathways. For example, one study have reported that the face-selective response in FG is earlier than that in the IOG[59], and the FG and STS are properly activated by faces despite the IOG lesions [60].

Several issues remain unaddressed in this study. First, the regions identified in this study showed clear face-selective activation, but they may not necessarily be dedicated to face perception; instead, some of them are likely involved in general cognitive functions, such as inferring others’ intention[10], [61], accessing knowledge about others [22], [24], or directing one’s own attention to objects and events that others are looking at [10], [11]. They are simply automatically recruited to act in concert with the face-selective regions when faces are presented. Second, the face-selective regions identified in the study are not exhaustive since the activation of a region is determined by many factors, such as the context of semantic information, information from different sensory modalities, and task requirements. That is, more regions are likely to be identified when other types of tasks or stimuli are used (e.g., tasks concerning facial expressions or gender) [48], [62]. Accordingly, the hierarchical structure may be changed to reflect the intrinsic properties of cognitive processes specified by tasks and stimuli. Future work is needed to examine how the face-processing network dynamically adjusts its weights among constituent nodes under different computational demands. Third, the functional connectivity analysis is not able to demonstrate how information flows within the network. Effective connectivity approaches, such as the dynamic causal model, may provide additional information on the face-processing network.

## Supporting Information

Figure S1 **Face-selective activation map from random-effect group analysis.** The activation map is generated by a general linear model with the contrast of faces versus objects from each participant as input and then models the variability between participants as a random effect. Color bar indicates the z-score from the contrast of faces versus objects in the group analysis. L: left hemisphere; R: right hemisphere.
(TIF)

Figure S2 **Thirty-seven group-level ROIs coded in different colors.** The ROIs are widely distributed across the brain, and together they capture 63.9% of total face-selective activation in all participants. The ROIs are labeled in random-rainbow color. The z coordinate increases 2 mm per slice from the upper left corner (z = −36) to the lower right corner (z = 70).
(TIF)

Figure S3 **Five exemplar GSS ROIs at the individual level from a representative participant.** From top to bottom, the ROIs are FG, IOG, STS, MFG and aFP. The group-level ROIs are outlined in blue and the subject-specific activation is shown in red.
(TIF)

Figure S4 **Percent BOLD signal changes for the 37 ROIs.** (A) The BOLD response for faces, objects, scenes and scrambled objects in the 25 ROIs that met the three criteria (i.e., cross-subject reliability, cross-session reliability and face selectivity). (B) The 12 ROIs that failed to meet at least one of the criteria. The y-axis indicates the percent BOLD signal change for each condition relative to the baseline condition (i.e., fixation). Error bars denote standard error of the mean.
(TIF)

Figure S5 Distribution of the 25 group-level ROIs that showed reliable face-selective activation across participants and across sessions. The ROIs are labeled in random-rainbow color. The z coordinate increases 4 mm per slice from the upper left corner (z = −28) to the lower right corner (z = 52).
(TIF)

Figure S6 **Dendrogram from the hierarchal clustering analysis based on anatomical distance between face-selective ROIs.** The anatomical distance between a pair of ROIs is calculated as the Euclidean distance between the peak coordinates of the ROIs. The dendrogram is generated in the same manner as the dendrogram based on functional connectivity.
(TIF)
