# Supplementary figures and images for: The Hierarchical Brain Network for Face Recognition
Source: PLoS One. 2013 Mar 20;8(3):e59886. doi: 10.1371/journal.pone.0059886 (PMC3603994; doi:10.1371/journal.pone.0059886)

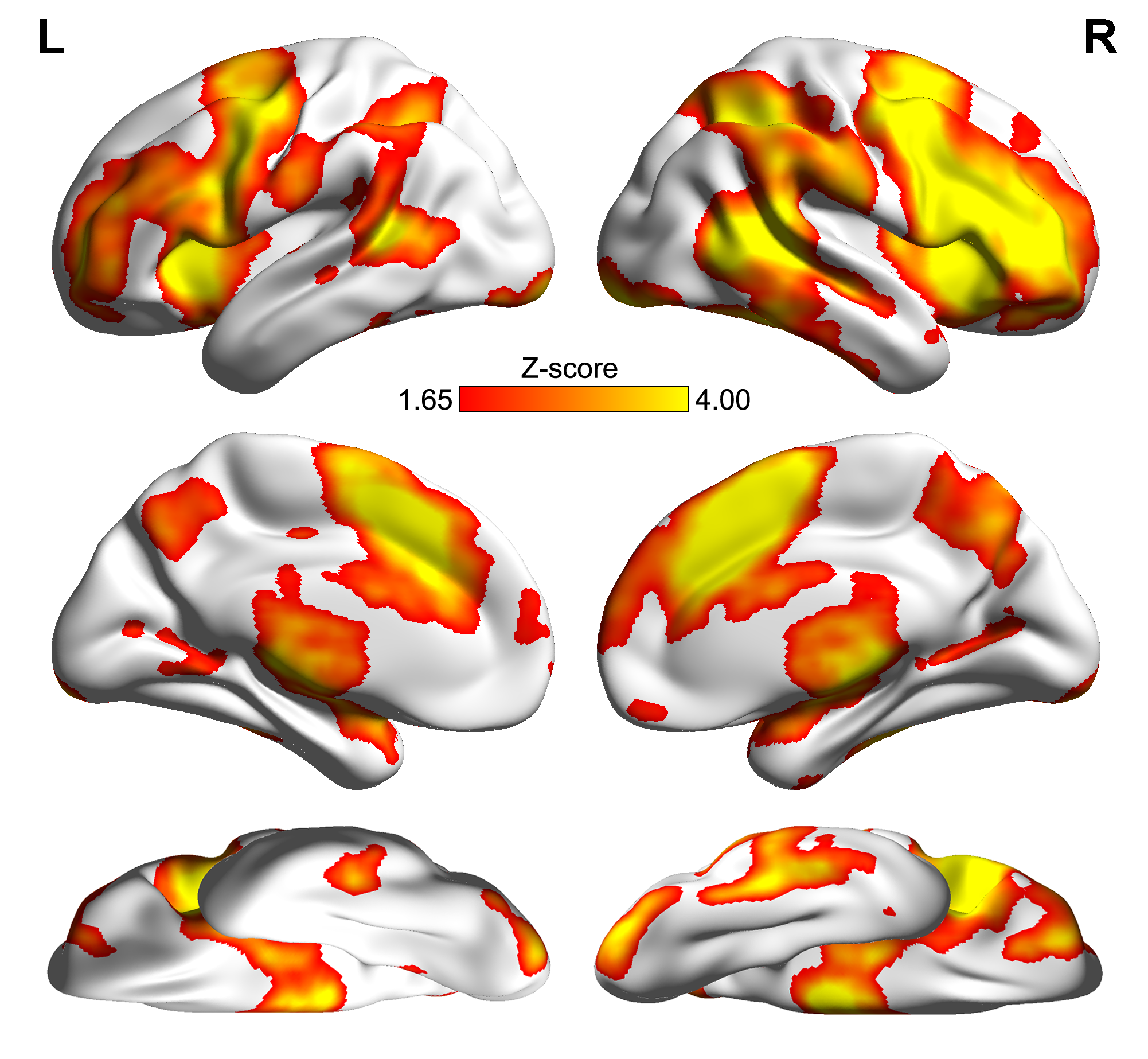

Supplement: Figure S1 — Face-selective activation map from random-effect group analysis. The activation map is generated by a general linear model with the contrast of faces versus objects from each participant as input and then models the variability between participants as a random effect. Color bar indicates the z-score from the contrast of faces versus objects in the group analysis. L: left hemisphere; R: right hemisphere. (TIF) [file pone.0059886.s001.tif]

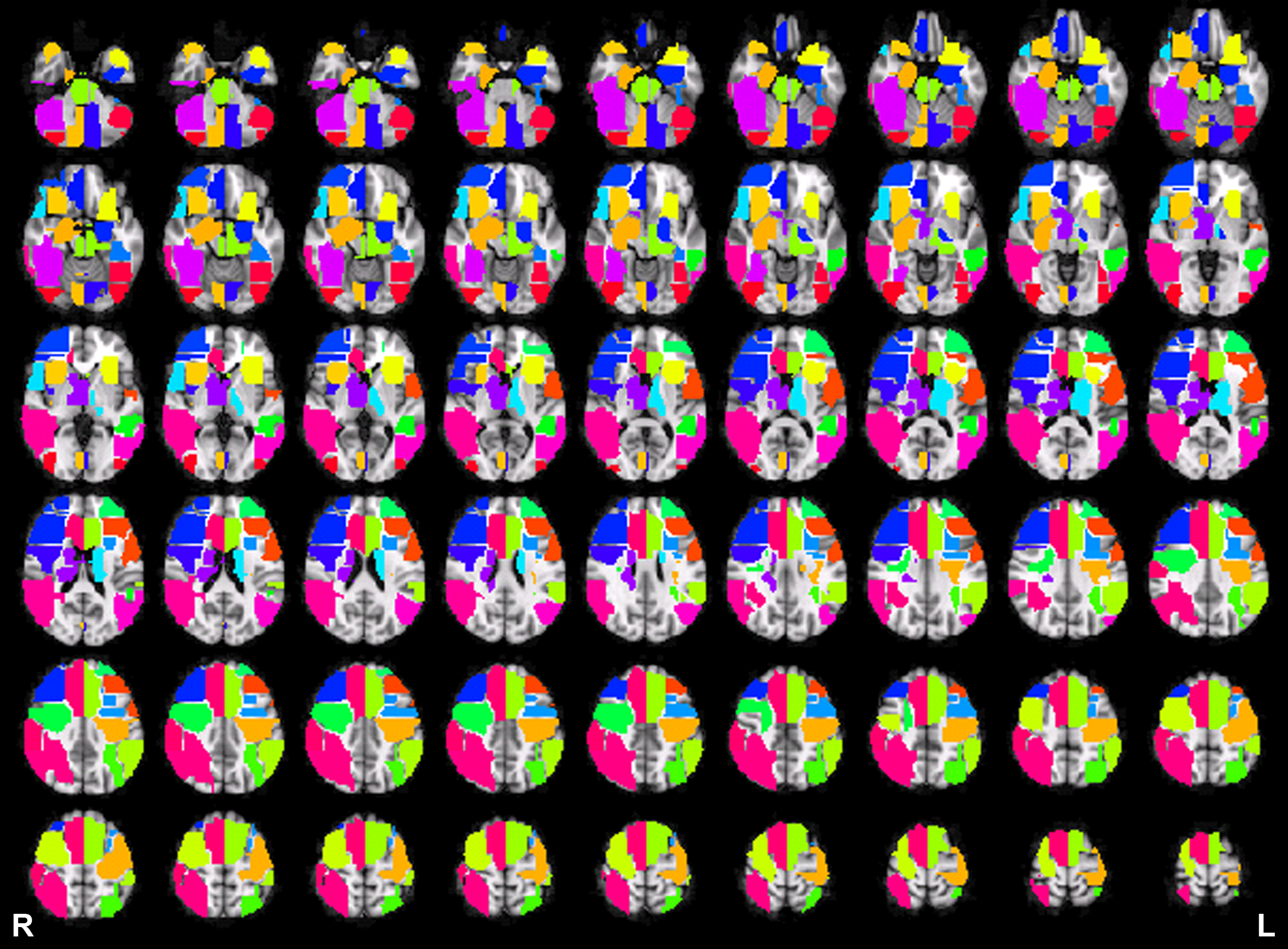

Supplement: Figure S2 — Thirty-seven group-level ROIs coded in different colors. The ROIs are widely distributed across the brain, and together they capture 63.9% of total face-selective activation in all participants. The ROIs are labeled in random-rainbow color. The z coordinate increases 2 mm per slice from the upper left corner (z = −36) to the lower right corner (z = 70). (TIF) [file pone.0059886.s002.tif]

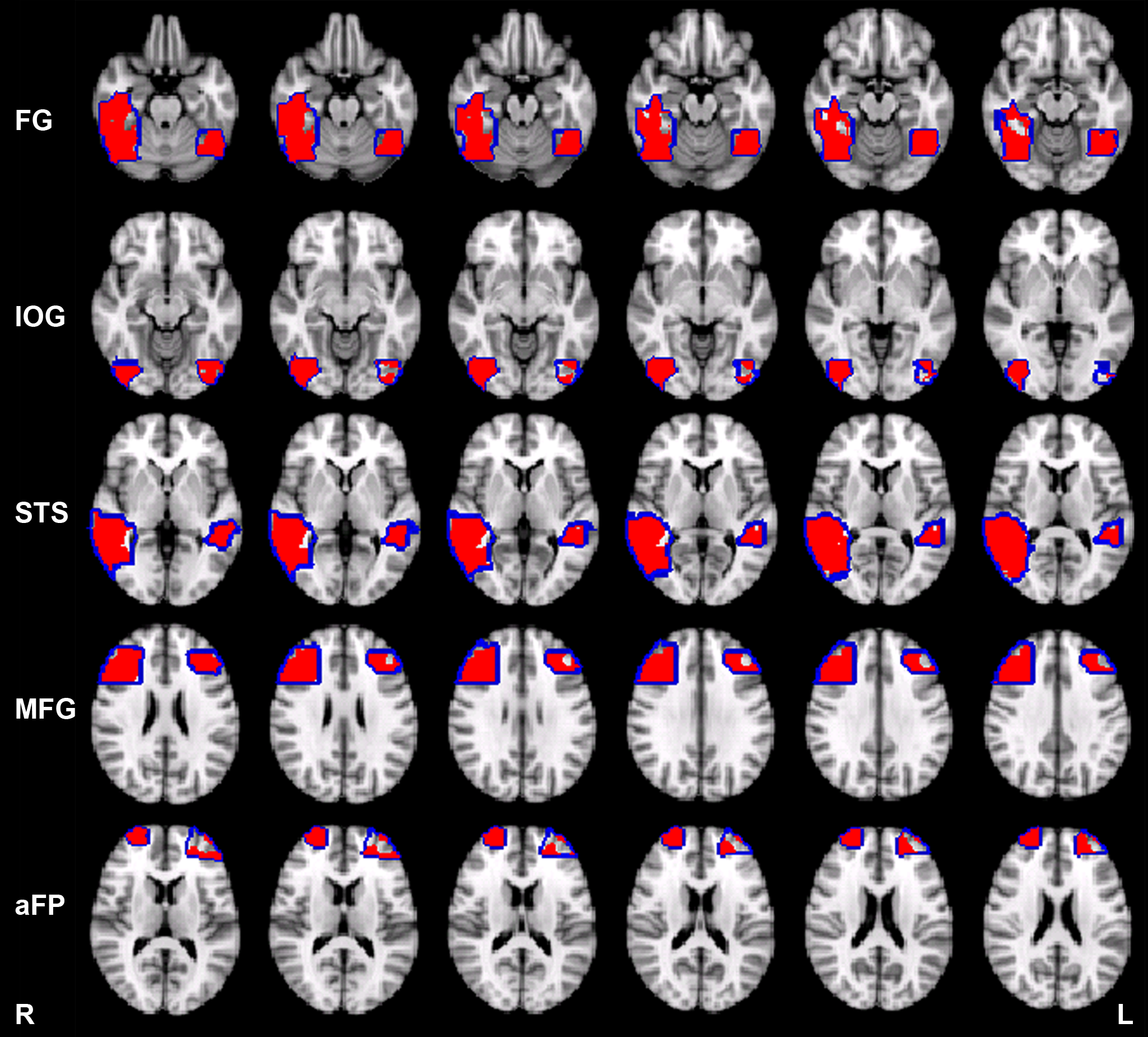

Supplement: Figure S3 — Five exemplar GSS ROIs at the individual level from a representative participant. From top to bottom, the ROIs are FG, IOG, STS, MFG and aFP. The group-level ROIs are outlined in blue and the subject-specific activation is shown in red. (TIF) [file pone.0059886.s003.tif]

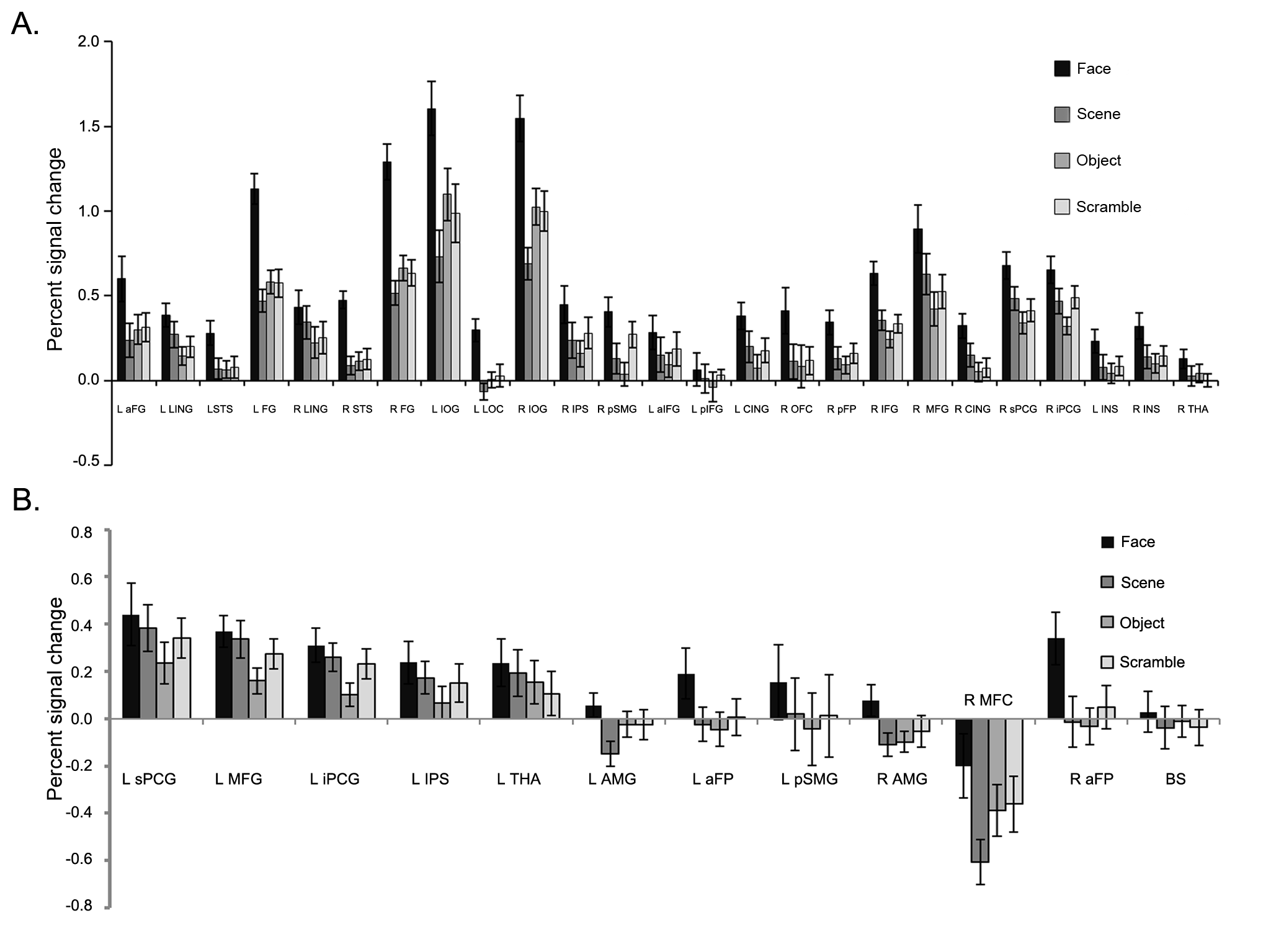

Supplement: Figure S4 — Percent BOLD signal changes for the 37 ROIs. (A) The BOLD response for faces, objects, scenes and scrambled objects in the 25 ROIs that met the three criteria (i.e., cross-subject reliability, cross-session reliability and face selectivity). (B) The 12 ROIs that failed to meet at least one of the criteria. The y-axis indicates the percent BOLD signal change for each condition relative to the baseline condition (i.e., fixation). Error bars denote standard error of the mean. (TIF) [file pone.0059886.s004.tif]

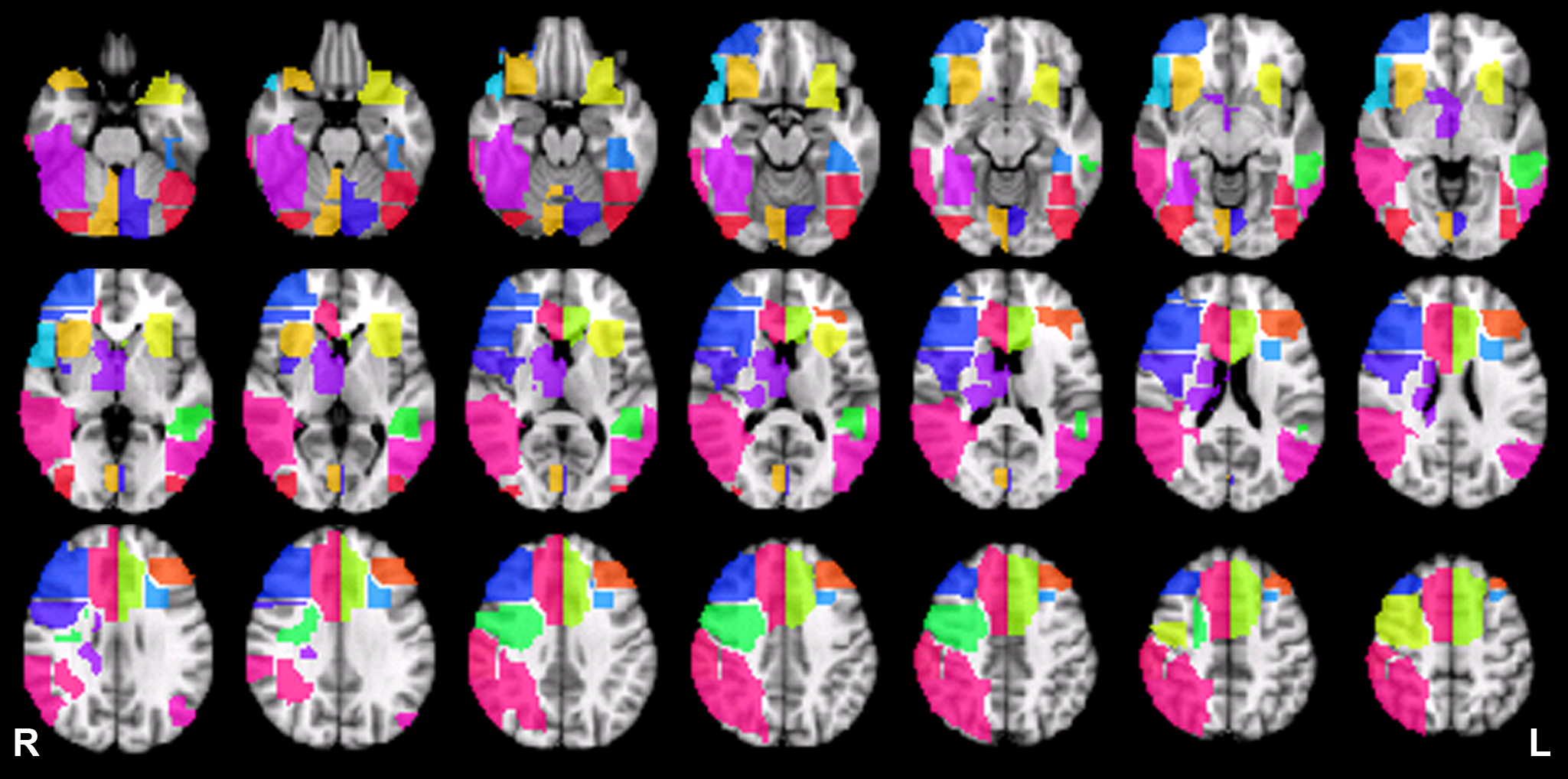

Supplement: Figure S5 — Distribution of the 25 group-level ROIs that showed reliable face-selective activation across participants and across sessions. The ROIs are labeled in random-rainbow color. The z coordinate increases 4 mm per slice from the upper left corner (z = −28) to the lower right corner (z = 52). (TIF) [file pone.0059886.s005.tif]

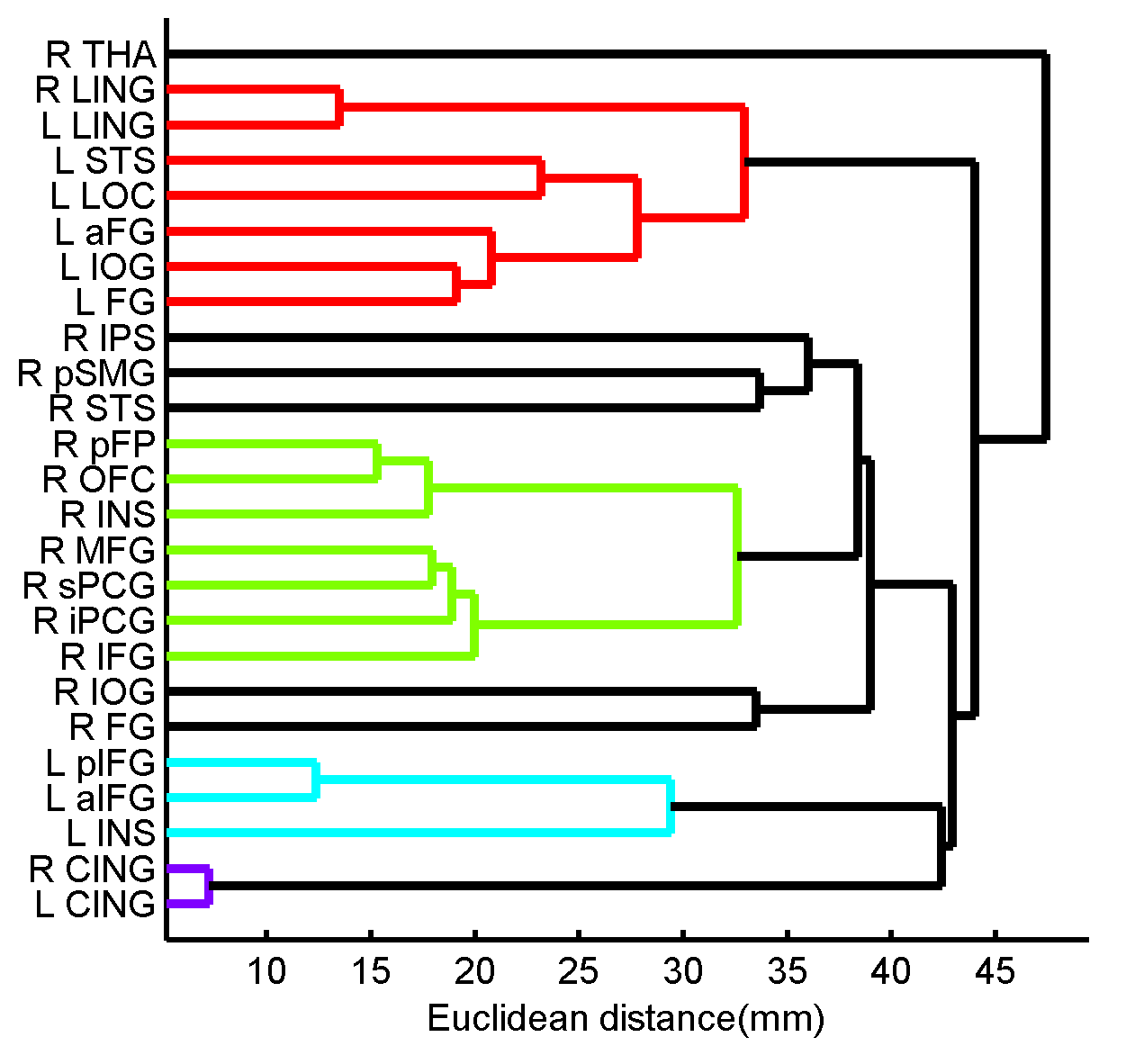

Supplement: Figure S6 — Dendrogram from the hierarchal clustering analysis based on anatomical distance between face-selective ROIs. The anatomical distance between a pair of ROIs is calculated as the Euclidean distance between the peak coordinates of the ROIs. The dendrogram is generated in the same manner as the dendrogram based on functional connectivity. (TIF) [file pone.0059886.s006.tif]
